# The Cancer Genome Atlas (TCGA) based m^6^A methylation-related genes predict prognosis in hepatocellular carcinoma

**DOI:** 10.1080/21655979.2020.1787764

**Published:** 2020-07-07

**Authors:** Jun Liu, Guili Sun, Shangling Pan, Mengbin Qin, Rong Ouyang, Zhongzhuan Li, Jiean Huang

**Affiliations:** a Department of Gastroenterology, The Second Affiliated Hospital of Guangxi Medical University, Nanning, China; b Department of Gastroenterology, The Fourth Affiliated Hospital of Guangxi Medical University/Liu Zhou Worker’s Hospital, Liuzhou, China; c Departments of Pathophysiology, Guangxi Medical University, Nanning, P.R. China

**Keywords:** Hepatocellular Carcinoma, *N*^6^-methyladenosine methylation, TCGA

## Abstract

The current study aims to investigate the significance of *N*^6^-methyladenosine (m^6^A) methylation-related genes in the clinical prognosis of hepatocellular carcinoma (HCC) using bioinformatics analyses based on The Cancer Genome Atlas (TCGA) database. Transcriptome data and corresponding clinical data on m^6^A methylation-related genes (including 15 genes) were obtained from TCGA database. Differential expression of 15 genes was identified. Survival curves of subgroups based on m^6^A methylation-related gene expression levels were plotted. We selected potential predictive genes and analyzed their prognostic values using bioinformatics methods. Eleven genes (*METTL3, YTHDF1, YTHDF2, YTHDF3, YTHDC1, YTHDC2, FTO, KIAA1429, HNRNPC, HNRNPA2B1*, and *RBM15*) were found to be overexpressed in HCC. Of these, five genes had worse survival (P < 0.05). There was a significant difference in the survival rate between subgroups with different expression levels of m^6^A. We selected five potential predictors (*METTL3, KIAA1429, ZC3H13, YTHDF1*, and *YTHDF2*) that met the independent predictive value. ZC3H13 was upregulated in patients with high cancer risk, whereas *METTL3, KIAA1429, YTHDF1*, and *YTHDF2* were downregulated. In summary, we found that the expression levels of m^6^A methylation-related genes were different in patients with HCC and correlated with survival and prognosis. This implies that m^6^A methylation-related genes may be promising prognostic indicators or therapeutic targets for HCC.

## Graphical abstract

**Figure:**
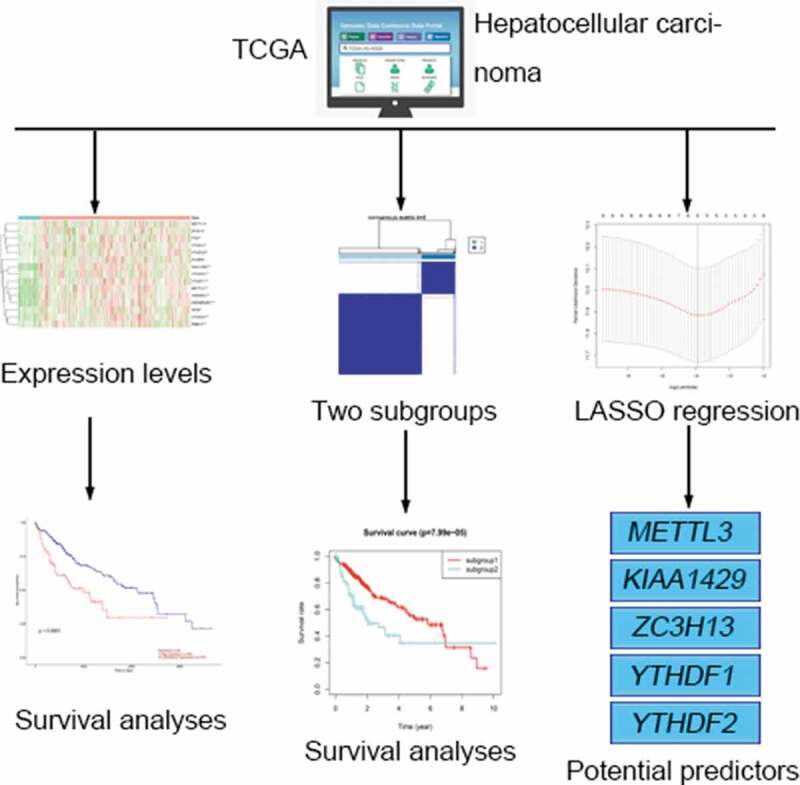

## Introduction

Hepatocellular carcinoma (HCC) is the most common primary form of liver cancer. In the past few decades, HCC has become the fifth most common cancer with a second highest mortality rate and a poor survival outcome worldwide [1]. Several risk factors have been linked to HCC [2]; however, its prognostic predictions are yet to be fully elucidated.

RNA modification is a post-transcriptional regulation that influences RNA stability and degradation. More than 150 RNA modifications have been identified, which are widely distributed in various types of RNA, such as mRNA, tRNA, rRNA, sncRNA, and lncRNA. However, the biological value of most RNA modifications remain unexplored due to technical limitations. *N*^6^-methyladenosine (m^6^A) which refers to the methylation modification of the sixth nitrogen (N) atom of adenine (A) accounts for more than 60% of RNA modifications and affects almost all RNA metabolic activities, such as splicing, transport, translation, and degradation [3].

The modification level of transcript m^6^A is dynamically regulated by methyltransferase (writer), binding protein (reader), and demethylase (erasers). *METTL3, METTL14, KIAA1429, RBM15, WTAP*, and *ZC3H13* have been shown to act as m^6^A methyltransferases (‘writers’) [4,5]. *HNRNPC, HNRNPA2B1*, and YT521-B homology (YTH) domain family members, including *YTHDC1, YTHDC2, YTHDF1, YTHDF2, YTHDF3* have been identified as the ‘readers’ of m^6^A and modulate mRNA metabolic activities [6–8].*ALKBH5* and *FTO*, key demethylases specifically removing m^6^A from target mRNAs, have been identified as the ‘erasers’ of m^6^A [9,10].

m^6^A affects multiple aspects of mRNA metabolism and regulates gene translation. Dysregulation of m^6^A is thus assumed to be relate to various biological processes, including cancer progression. Moreover, these genes do not function alone, but can interact with each other. However, whether m^6^A and its key modulators play a specific role in inhibiting or promoting cancers remain inconclusive to date. Although great progress has been made in finding biomarkers for tumor prognosis, less than 1% of biomarkers are used in clinical practice [11]. Furthermore, most investigations on m^6^A methylation-related genes are single or small combination studies. At present, there is no comprehensive study on the role of m^6^A methylation-related genes in HCC. Therefore, it is important to assess the relationship between m^6^A methylation-related genes and HCC at the genetic level. The present study analyzed associations between m^6^A methylation-related gene expression and clinical prognosis of patients, in an attempt to find novel prognostic biomarkers and therapeutic targets for HCC.

### Methods and materials

#### Data collection

Transcriptome data and corresponding clinical data of HCC were obtained from The Cancer Genome Atlas (TCGA) database (https://portal.gdc.cancer.gov/).mRNA expression data of 374 tumors and 50 normal tissue, as well asclinical information including age, gender, grade, clinical stage, and TNM stage of 348 HCC patients were collected.

#### Bioinformatic analysis/statistics

The differential expression of 15 m^6^A methylation-related genes (*METTL3, METTL14, WTAP, YTHDF1, YTHDF2, YTHDF3, YTHDC1, YTHDC2, FTO, KIAA1429, ALKBH5, FTO, HNRNPC, HNRNPA2B1*, and *RBM15*) in HCC and normal control samples were evaluated using R software (Version 3.8; http://www.bioconductor.org/packages/release/bioc/html). The heatmap of these genes were plotted using R software. Pearson correlation analyses were performed to identify gene-to-gene correlation. Kaplan–Meier (KM) survival analysis was performed to assess the effect of each gene on survivalbased on UALCAN website (http://ualcan.path.uab.edu/index.html). The P-value of 0.05 was considered the significant threshold in all tests.

#### PCA and survival analyses of subgroups

Consensus clustering is a class discovery technique for the detection of unknown possible clusters consisting of items with similar intrinsic features [12]. Based on comprehensive expression of the 15 genes, we identified distinct subgroups of 374 tumor samples with R’s ConsensusClusterPlus package, using principal component analysis (PCA) to verify the results of the grouping. Survival curves between two subgroups were plotted using the KM method.

#### Prognostic value of m^6^A methylation-related genes

The 15 genes in question were analyzed by univariate Cox regression, in which candidate genes were selected if they satisfied the screening condition of P < 0.05. Thereafter, we utilized LASSO regression for high-dimensional data to select the most useful prognostic factors using the ‘glment’ package in R software [13]. Five genes were selected and their related risk score were also calculated. Patients were divided into high-risk and low-risk groups based on the median expression of m^6^A methylation-related genes. The relationship between m^6^A related genes and survival rates was analyzed by the KM survival approach. Log-rank tests were employed to calculate the P-value of KM survival curves. The receiver operating characteristic (ROC) curve was drawn to test the accuracy of the model. Univariate and multivariate Cox regression analyses were performed to identify prognostic factors for HCC. The heatmap of m^6^A methylation-related genes and clinical risk factors were plotted.

## Results

### TCGA dataset and patients’ characteristics

HCC tissue (374) and 50 adjacent normal tissue from TCGA were enrolled in the current study. A total of 348 patients (238 males and 110 females) were included following exclusion of samples having incomplete clinical data. The average age of patients was 58.80 years. Clinical data included age, gender, grade, clinical stage, and TNM stage.

### Expression of m^6^A methylation-related genes in HCC

**Figure 1.**
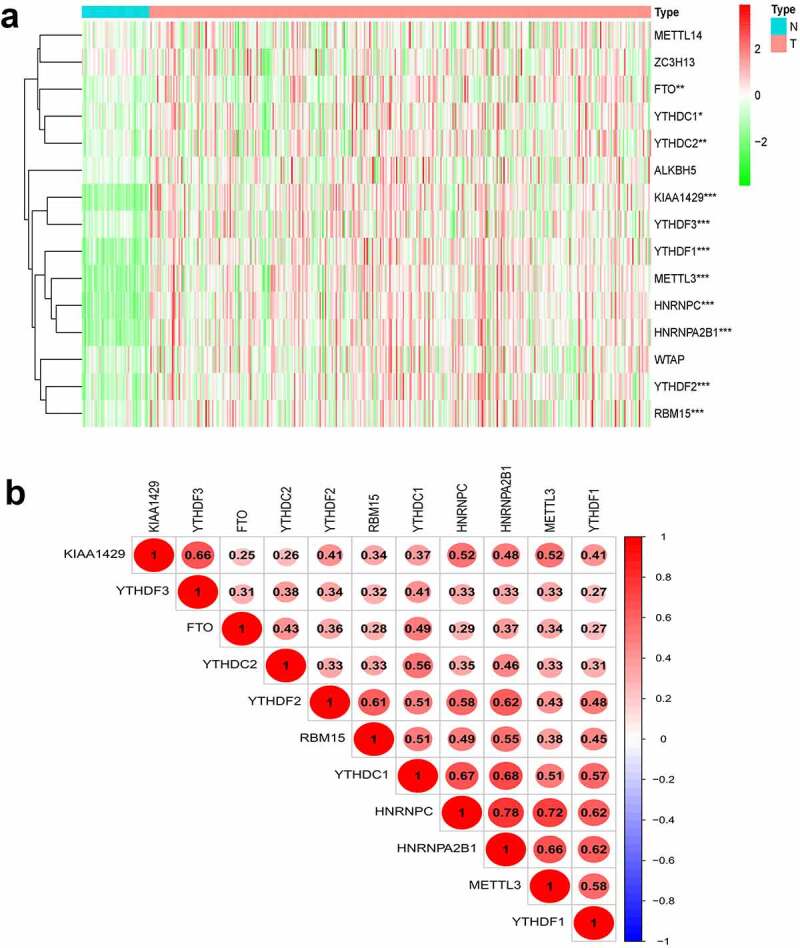
Expression, correlation, and prognostic information of m^6^A methylation-related genes. (a) Heatmaps of m^6^A methylation-related genes expressed in tumors and adjacent normal tissue. (***P < 0.001, ** P < 0.01, *P < 0.05) (b) Correlation matrix of interaction in m^6^A methylation-related genes. Correlation coefficients are plotted with negative correlation (blue) and positive correlation (red).

We constructed a gene expression heatmap with 15 m^6^Amethylation-related genes to get an overview for the expression in HCC, according to TCGA, 11 genes (*METTL3, YTHDF1, YTHDF2, YTHDF3, YTHDC1, YTHDC2, FTO, KIAA1429, HNRNPC, HNRNPA2B1*, and *RBM15*) showed significant upregulation in tumors compared with that in adjacent normal tissue in HCC (Figure 1(a)).

As shown in Figure 1(b), Pearson correlation analysis demonstrated that all 11 genes showed a positive correlation with each other. The highest correlation was observed for *HNRNPC* and *HNRNPA2B1* with a correlation coefficient of 0.78. *HNRNPC* and *METTL3* as well as *YTHDC1* and *HNRNPA2B1* were also strongly correlated, and their correlation coefficients were 0.72 and 0.68, respectively.

### Survival analysis of m^6^A methylation-related genes

**Figure 2.**
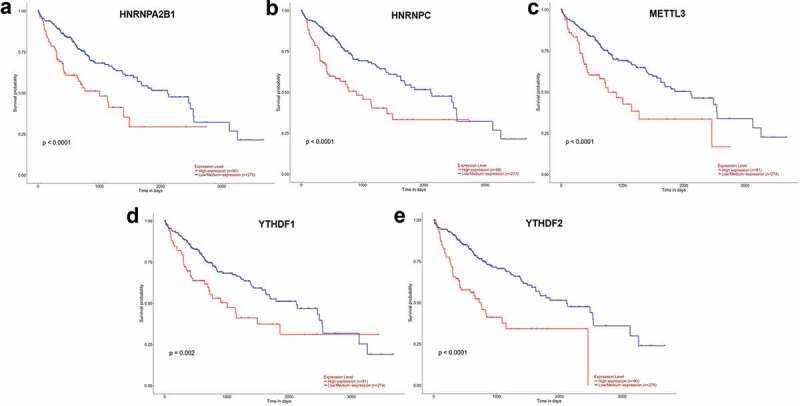
(a–e) Prognostic information for five of 11 genes, which had a significantly worse survival rate (P < 0.05).

UALCAN (http://ualcan.path.uab.edu/index.html) online tool was used to identify survival data of 11 genes. It was found that patients with high expression of five genes (*HNRNPA2B1, HNRNPC, METTL3, YTHDF1*, and *YTHDF2*) had a significantly worse survival (P < 0.05) (Figure 2(a–e)) while the remaining six genes showed no significant difference (P > 0.05).

**Figure 3.**
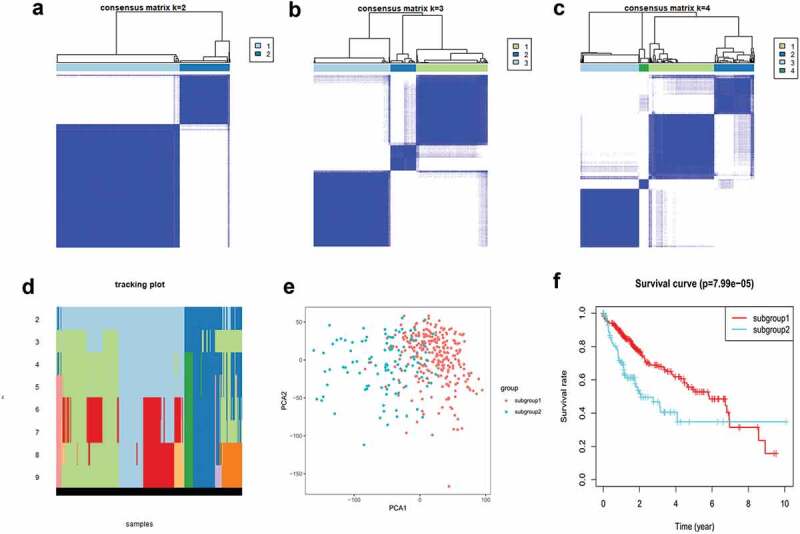
Identification and analysis of two subgroups of 374 tumor samples that exhibited distinct m^6^A expression. (a–c) Consensus clustering matrix for k = 2, 3, and 4. (d) Tracking plot for k = 2 to 9. (e) Principal component analysis (PCA) of the two subgroups. (f) Kaplan–Meier survival plots of the two subgroups.

Based on m^6^A methylation-related gene expression levels, we identified distinct subgroups of 374 tumor samples using R’s ConsensusClusterPlus package. And we calculated cluster-consensus and item-consensus results. The output displayed k (2 to 4) subgroups, shown in Figure 3(a–c). We found that k = 2 achieved adequate selection. All patients were successfully categorized into two subgroups in terms of the most stable k value (Figure 3(a–d)).

As shown in Figure 3(e), subgroup1 represented a high level of gene expression, while subgroup2 did not. The horizontal axis represents the first principal component, while the vertical represents the second. Principal component analysis (PCA) showed that subgroup1 can assemble together and so can subgroup2. These results indicated that our grouping was accurate. Overall survival analysis of differentially expressed genes indicated that survival duration of subgroup1 significantly improved (P < 0.05) (Figure 3(f)).

### Construction of LASSO model

**Figure 4.**
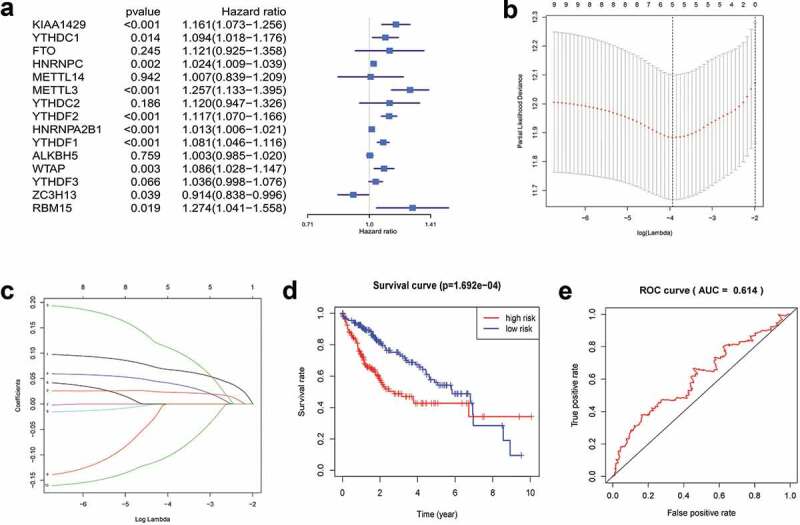
Gene selection and survival analysis in HCC prognosis prediction. (a) Forest plots for hazard ratios (HRs) of survival‑associated m^6^A methylation-related genes in HCC. (b) Partial likelihood deviance versus log (λ)was drawn using LASSO Cox regression model. (c) Coeﬃcients of selected features are shown by lambda parameter. (d) Kaplan–Meier survival plots of the two groups. (e) ROC curves of the survival model in HCC (AUC = 61.4%).

We used univariate Cox regression to analyze 15 genes, and 10 candidate genes were selected with P < 0.05 as a screening condition (Figure 4(a)). The LASSO Cox regression model was used to select the most predictive genes as prognostic indicators. λ was selected when the median of the sum of squared residuals was the smallest. Five potential predictors (Figure 4(b–c)). *METTL3, KIAA1429, ZC3H13, YTHDF1*, and *YTHDF2* were identified as prognostic factors for HCC. The risk score of five genes was also calculated for further univariate and multivariate Cox regression analyses.

Patients were divided into high-risk and low-risk groups based on the combined model with cutoff values at the median expression of the five candidate genes. The low-risk group showed consistent better prognosis than high-risk groups. The survival curve was plotted by the KM method (Figure 4(d)). We also compared the prognostic efficiency of risk factors through ROC curves. The results showed that areas under the curve (AUC) were 61.4% (Figure 4(e)), indicating that m^6^A methylation-related genes could serve as biomarkers in prognosis of HCC.

### Prognostic value of the 5 m^6^A methylation-related genes

**Figure 5.**
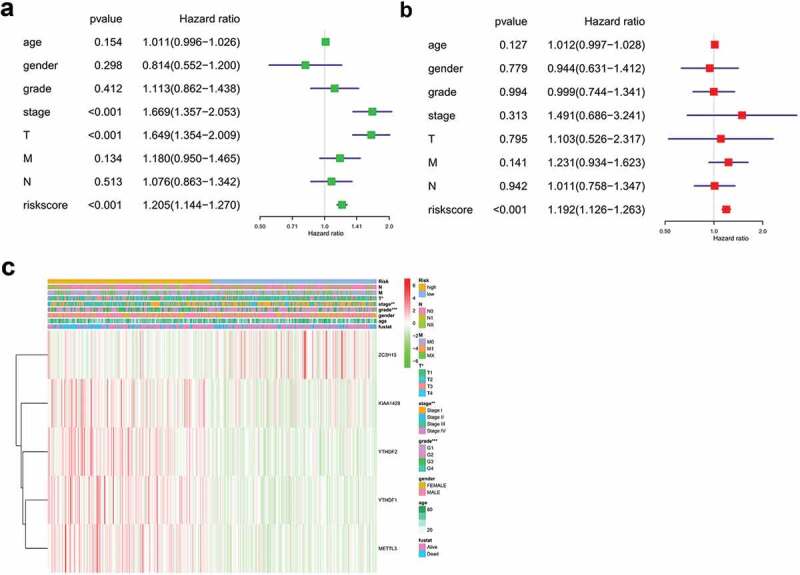
Forest plot and heatmap of m^6^A methylation-related genes and clinical risk factors. (a) Forest plot of univariate Cox regression analysis in HCC. (b) Forest plot of multivariate Cox regression analysis in HCC. (c) Heatmap of m^6^A methylation-related genes and clinical risk factors.(***P < 0.001, ** P < 0.01, *P < 0.05).

Univariate analysis showed that T stage, clinical stage, grade, and risk score of m^6^A methylation-related genes affected the prognosis of patients (P < 0.05). Age, gender, grade, M stage, and N stage did not correlate with the prognosis of HCC (P > 0.05) (Figure 5(a)). The results of multivariate regression analysis showed that the risk score of m^6^A methylation-related genes was an independent prognostic factor in HCC (P < 0.05) (Figure 5(b)).

As shown in Figure 5(c), protective genes*ZC3H13* had a tendency to be upregulated in low-risk patients, whereas *METTL3, YTHDF1, YTHDF2*, and *KIAA1429* had a tendency to be highly expressed in high-risk patients. T stage, clinical stage, and grade were linked with the degree of risk, while M stage, N stage, gender, and age had no significance (P > 0.05).

## Discussion

Hepatocellular carcinoma has become a fundamental public health concern worldwide. To identify more useful prognostic biomarkers for HCC, using bioinformatics based on TCGA, we established 15 gene signatures (including *METTL3, METTL14, KIAA1429, RBM15, ZC3H13, WTAP, YTHDF1, YTHDF2, YTHDF3, YTHDC1, YTHDC2, HNRNPC, HNRNPA2B1, ALKBH5*, and *FTO*) for HCC prognosis prediction. Eleven m^6^A methylation-related genes were up-regulated in HCC and all showed a positive correlation with each other. Five genes (*HNRNPA2B1, HNRNPC, METTL3, YTHDF1*, and *YTHDF2*) were linked to significantly worse survival. Our study demonstrated that m^6^A methylation-related genes were widely distributed in tumor tissue, indicating their important roles in HCC prognosis prediction. In addition, m^6^A methylation-related genes were strongly associated with each other in regulatory networks, suggesting their cooperation in cancer development. Furthermore, *HNRNPA2B1, HNRNPC, METTL3, YTHDF1*, and *YTHDF2* may have deleterious effects on patients with HCC due to their association with worse survival. These findings imply that m^6^A modulators are potential targets for HCC treatment. In the following paragraphs, we will briefly discuss the relationship between these genes and HCC one by one.

Consensus cluster uses a variety of different clustering methods as inputs, so as to find a more suitable clustering method than each individual method. Subgrouping tumors helps to develop personalized treatments for individual patients. Based on gene expression levels, data were divided into two subgroups using R’s ConsensusClusterPlus package. Principal component analysis showed a separation between subgroup1 and subgroup2. Overall survival analysis indicated that survival duration of subgroup1 significantly improved, suggesting that survival time correlated with comprehensive expression level of m^6^A methylation-related genes.

The LASSO algorithm analyzes all independent variables simultaneously and selects the most influential variables [14]. Thus, far more accurate than the traditional regression methods. According to LASSO Cox analysis, five of 15 genes (*METTL3, YTHDF1, YTHDF2, KIAA1429*, and *ZC3H13*) were identified as prognostic factors for HCC. The predictive power of m^6^A methylation-related genes on HCC prognosis was evaluated by the ROC curve. The results demonstrated that m^6^A methylation-related genes were involved in the survival of HCC. The risk score of m^6^A methylation-related genes (*METTL3, YTHDF1, YTHDF2, KIAA1429*, and *ZC3H13*) might be a powerful biomarker for HCC survival. High expression levels of *METTL3, YTHDF1, YTHDF2*, and *KIAA1429* predicted a poor prognosis, whereas *ZC3H13* can be regarded as protective genes. T stage, clinical stage, and grade were linked with the degree of risk. However, precise biological behaviors of these five genes in HCC remain to be interpreted. We conducted a comprehensive biological analysis on the 15 most important m^6^A methylation-related genes, which was more comprehensive than previous studies on the influence of a single gene on disease. As interactions between m^6^A methylation-related genes exist, our study more accurately reflected their influence on HCC.

Heterogeneous nuclear ribonucleoprotein A2B1 (*HNRNPA2B1*) is an m^6^A reader which promotes miRNA biogenesis. Previous studies showed that *HNRNPA2B1* was highly expressed in a variety of human cancers, such as prostate cancer [15], pancreatic cancer [16], and hepatocellular carcinoma [17]. Higher expression levels of *HNRNPA2B1* have been reported in HCC [18]. Zhou et al. [19] reported that *HNRNPAB* induces epithelial–mesenchymal transition (EMT) and promotes metastasis of hepatocellular carcinoma, which was consistent with our findings.

Heterogeneous nuclear ribonucleoprotein C (*HNRNPC*) is an RNA-binding protein and well known for its regulatory roles in RNA splicing, 3′ end processing [20], and translation [21]. Overexpression of *HNRNPC* has been observed in multiple tumors, including glioblastomas [22], melanomas [23], and hepatocellular carcinomas [24]. However, the role of *HNRNPC* in HCC is still poorly documented. Our study provided a reference for further research.

Methyltransferase-like 3 (*METTL3*) determines the levels and distribution of target-specific m^6^A modifications [25]. Knockdown of *METTL3* remarkably reduced the level of m^6^A in mRNAs^3^. *METTL3* has been demonstrated to participate in tumorigenesis and the progression of several cancers [26]. For example, *METTL3* promotes the progression of breast cancer by inhibiting tumor suppressor let-7 g [27]. Visvanathan et al. reported that upregulation of *METTL3* was associated with worse survival in glioblastoma cells [28]. Chen et al. reported that *METTL3* served as an oncogene and contributed to the progression of HCC and lung metastasis [29]. Upregulation of *METTL3* contributed to cancer metastasis and predicted poor prognosis in patients with HCC [30]. However, Aravalli et al. observed that knockout of *METTL3* remarkably suppressed HCC tumorigenesis and development [31]. The main reason for the dual role of *METTL3* in cancer regulation may account for different targeted pathways and cancer heterogeneity.

YTH domain family 1 and 2 (*YTHDF1*and *YTHDF2*) are located in the cytoplasmic compartment [32,33]. YTHDF1 interacts with translation initiation factors to promote translation.*YTHDF2* regulates the stability of target mRNAs [34]. The binding of ribosome to m^6^A-modified RNA and the translation of RNA can be reduced by knocking down *YTHDF1* [35]. Previous studies have demonstrated that *YTHDF1* and *YTHDF2* are highly expressed in hepatocellular carcinoma, affecting cell cycle and metabolism of tumor cells, and the prognosis of high *YTHDF1* expression in patients was poor [36]. Our survival analysis results were in agreement with those of previous studies.

*KIAA1429* is a component of m^6^A ‘writers.’ Knocking down of *KIAA1429* led to a considerable reduction of m^6^A in mRNA, suggesting that *KIAA1429* was essential for the methyltransferase complex. Qian et al.found that *KIAA1429* was highly expressed in breast cancer tissue, but frequently down-regulated in non-cancerous breast tissue [37]. A previous study demonstrated that *KIAA1429* facilitated migration and invasion of HCC by inhibiting ID2 via upregulation of m^6^A modification [38]. These studies were consistent in that *KIAA1429* played an important role in cancer progression and might potentially prevent or treat cancers.

Zinc finger CCCH domain-containing protein 13 (*ZC3H13*) acts as m^6^A methyltransferases (‘writers’). It usually functions by interacting with other m^6^A writer complex subunits [39]. Knockdown of *ZC3H13* in mouse embryonic stem cells significantly decreases global m^6^A level on mRNA [40]. However, a paucity of evidence for liver malignancy research exists. Our study suggests that *ZC3H13* has a tendency to be upregulated in low-risk patients, indicating that the prognosis of HCC may be improved by regulating *ZC3H13* expression.

Overall, combination of m^6^A methylation-related genes with clinical parameters may have better predictive efficacy than a single biomarker. In recent years, m^6^A methylation-related genes have shown great potential in prognosis prediction of cancer. Our study preliminarily demonstrated that expression levels of m^6^A methylation-related genes play important roles in progression of HCC and may act as a prognostic predictor for this disease. However, there was a limitation to the present study. Our study was based on an individual source from TCGA, without validation from independent cohorts. More studies are needed for further clarification of these findings.

## Conclusion

The expression of m^6^A methylation-related genes highly correlates with clinical features of HCC and may predict its prognosis as well as guide individualized therapy in clinical practice. Our study provides important evidence for future detection of the role of m^6^A methylation in HCC.

## Data Availability

The datasets analyzed during the present study are available from The Cancer Genome Atlas (TCGA) database (https://portal.gdc.cancer.gov/).

## Highlights

1. The expression levels of m^6^A methylation-related genes were correlated with prognosis of HCC.
2. Five m^6^A methylation-related genes were upheld the independent predictive value of HCC.
3. m^6^A methylation-related genes can be regarded as prognostic indicators.
